# Low glenoid neck Hounsfield units predict patients at high risk of mechanical complication after total shoulder arthroplasty

**DOI:** 10.1016/j.jsea.2026.100001

**Published:** 2026-01-29

**Authors:** Patrick Sun, Cathleen Cahill, Darlington Nwaudo, Gregory S. Stacy, Lewis L. Shi, Nicholas H. Maassen

**Affiliations:** a Pritzker School of Medicine, University of Chicago, Chicago, IL, USA; b Department of Orthopaedic Surgery and Rehabilitation Medicine, University of Chicago Medicine, Chicago, IL, USA; c Department of Radiology, University of Chicago Medicine, Chicago, IL, USA

**Keywords:** Hounsfield unit, Glenoid, Total shoulder arthroplasty, Complication, Revision, Bone mineral density, Computed tomography

## Abstract

**Background:**

Computed tomography Hounsfield units (HUs) estimate bone mineral density, but their predictive value for total shoulder arthroplasty (TSA) mechanical complications is unknown. This study assessed whether glenoid HU (gHU) can be used to predict mechanical complication risk.

**Methods:**

In this retrospective cohort study at a single tertiary academic center (January 2011-April 2024), pre-operative computed tomography scans from 250 TSAs in 233 patients were independently reviewed by 3 interdisciplinary reviewers to measure gHU. Mechanical complications, including periprosthetic or intraoperative fractures and aseptic implant loosening, were identified and stratified by gHU. Inter-rater reliability was assessed with the intraclass correlation coefficient. Receiver operating characteristic analysis identified optimal gHU thresholds. Risk-group comparisons used chi-square tests and multivariable logistic regression.

**Results:**

Among 250 TSAs in 233 patients, intraclass correlation coefficient for gHU measurement was 0.90 (95% confidence interval 0.87-0.92). Receiver operating characteristic analysis yielded a cutoff of 177 HU (area under the curve 0.59; sensitivity 39%; specificity 81%). Mechanical complications occurred in 29% of cases with gHU <177 vs. 13% with gHU ≥177 (*P* = .008; adjusted *P* = .02). Patients with gHU <110 had the highest risk (57% vs. 14%; *P* < .001; adjusted *P* = .002).

**Conclusion:**

Pre-operative gHU measurements can help to predict increased risk of mechanical complications after TSA. A gHU threshold of 177 HU identifies patients at elevated risk, while gHU <110 marks the highest-risk group.

Total shoulder arthroplasty (TSA), both anatomic (aTSA) and reverse (rTSA), have long track records of considerable improvement in shoulder function and pain coupled with an acceptably low complication rate. The continued long-term success of these two variations of shoulder arthroplasty, along with the increasing indications for TSA has led to a large increase in overall shoulder arthroplasty procedures.^20^^,^^21^^,^^27^ It is estimated that over 820,000 people in the US were living with a TSA in 2017, with 60% of them being performed within the prior 5 years.^12^ Despite 10-year revision-free survival rates reported to be greater than 90%, the large and growing number of TSAs performed each year is driving an increase in post-operative complications and revision surgeries.^6^^,^^12^^,^^28^^,^^33^ It is estimated that over 10,000 revision surgeries are performed annually, at a cost of over $200 million to the US health care system.^12^ Pre-operatively identifying patients at higher risk of complication and subsequent revision can inform perioperative management, better educate patients to the risks of surgery, and possibly mitigate part of the cost and morbidity associated with complications after TSA.

The most common complications after TSA include instability (31.3% of all complications in rTSA; 10.1% in aTSA), infection (17.8% in rTSA; 4.9% in aTSA), component loosening (11.3% in rTSA; 39.1% in aTSA), and periprosthetic fracture (20.8% in rTSA; 6.7% in aTSA).^3^^,^^4^^,^^10^^,^^34^ Of these complications, component loosening and periprosthetic fractures have an association with patient low bone mineral density (BMD).^5^^,^^9^ The gold standard for diagnosis of low BMD is dual energy x-ray absorptiometry (DEXA) scans. However, DEXA scans are underutilized, with previous work showing that among Medicare beneficiaries over the age of 65, approximately 30% of women and 4.4% of men underwent DEXA screening.^8^ Many patients presenting to orthopedic clinics have not had formal work up for BMD and are unaware of any potential diagnosis of osteopenia or osteoporosis. The importance of bone quality for this and many other orthopedic surgeries cannot be understated but pre-operative DEXA scans on all patients is likely an unrealistic goal given limited health care resources and time.

Ideally, orthopedic surgeons could use information already obtained in their standard pre-operative work or planning for arthroplasty to also assess BMD. Pre-operative computed tomography (CT) scans have gained popularity for TSA due to improved understanding of anatomy and use in pre-operative planning. The Hounsfield unit (HU) is already obtained information that could be utilized in this manner. HUs measure the amount of radiation absorbed by different structures during CT scans and quantify tissue density. Previous work has demonstrated that abnormal BMD can be predicted from HU measured from CT scans. Nappo et al^22^ demonstrated that glenoid metaphyseal bone HU can be predictive, in the general population, of risk for having BMD abnormality. HUs have also been utilized to directly predict the risk of complications after surgery, including spinal instrumentation for metastatic cancer and total ankle arthroplasty (TAA).^1^^,^^2^^,^^7^^,^^11^^,^^13^, ^14^, ^15^, ^16^^,^^22^, ^23^, ^24^, ^25^, ^26^^,^^29^, ^30^, ^31^, ^32^^,^^35^^,^^36^ Within the shoulder literature, low HU have been associated with higher risk of proximal humerus fracture.^18^ However, to date, there has not been any investigation on whether glenoid Hounsfield unit (gHU) can be used to predict the risk of mechanical complications after TSA.

In this study, we aim to characterize the ability of gHU to predict mechanical complications after TSA using a retrospective cohort. Secondarily, we aim to determine the ability of gHU to predict abnormal BMD.

## Methods

### Data collection

**Figure 1:**
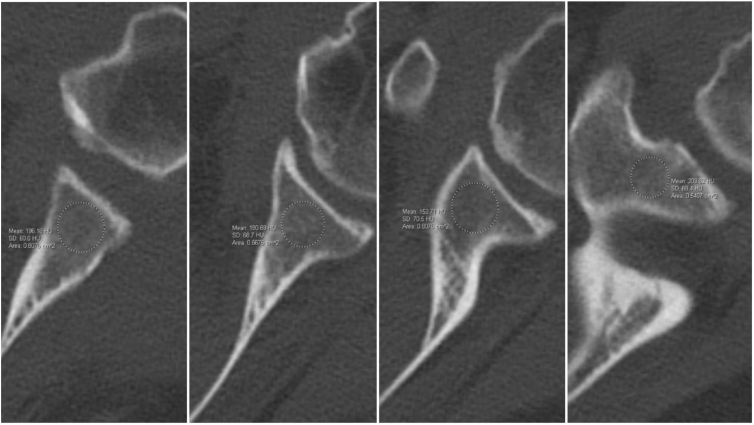
Measurement of HU from caudal to cranial (left to right) on computed tomography. *HU*, Hounsfield unit.

A random subset of patients that underwent aTSA or rTSA and had a pre-operative upper-extremity CT scan performed at a single academic center between April 2, 2011, and January 4, 2024, were reviewed retrospectively. Glenoid HU for each patient were determined independently by a senior orthopedic resident, a fellowship-trained shoulder and elbow surgeon and a fellowship trained musculoskeletal radiologist using previous validated methodology described in Nappo et al.^22^ Axial CTs of the glenoid were reviewed and the circle region-of-interest tool was utilized over the glenoid neck and HU measurements were produced using IMPAX software (Fig. 1). Reviewers ensured that the region sampled stayed within the anterior and posterior cortices, excluding the cortices themselves as well as any subchondral bone, sclerosis, or cysts. For each axial image, the total number of slices that traversed the glenoid neck were split into quarters, and the HU were measured at each of these intervals. Measurements were started at the caudal aspect of the glenoid neck and began one slice cranial to the most caudal slice that included the glenoid. The average value of the four slices through the glenoid neck was recorded as the average gHU for each CT scan. Reviewers were blinded to all patient outcomes, including the presence of mechanical complication and BMD status. Inter-rater reliability for CT scan measurements was calculated.

Subsequently, chart review was performed to collect demographic data and surgical outcomes. If available, BMD was determined by using the lowest t-score from DEXA scan data at the lumbar spine, femoral neck, and total femur. T-scores between −2.5 and −1 are designated as osteopenia and t-scores below −2.5 as designated as osteoporosis. Both osteoporosis and osteopenia were considered as abnormal BMD for this study. Fragility fracture was defined as a distal radius, proximal humerus, femoral neck, or lumbar spine fracture resulting from trauma approximately equivalent to or less than a fall from standing height.

**Figure 2:**
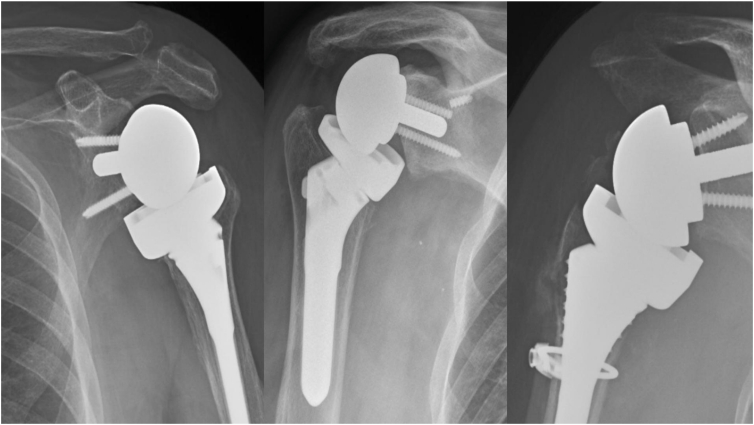
Adverse events included in definition of mechanical complication from left to right: acromial stress fracture, aseptic glenoid loosening, and intraoperative periprosthetic fracture.

The primary outcome measures were mechanical complications defined as periprosthetic fracture, intraoperative fracture, or aseptic loosening of either the glenoid or humeral implant. Examples of several complications are given in Figure 2. All primary outcome measures were adjudicated by a fellowship-trained shoulder and elbow surgeon who was blinded to gHU. Complications such as infection and dislocation were not considered for this study, as BMD is not a known risk factor for these specific complications. Secondary outcomes assessed correlation between gHU and revision, fragility fracture, and abnormal BMD based on previously reported thresholds. Correlation between these thresholds and complication rates were also assessed.

### Statistical analysis

Statistical analysis was performed using Excel and R. Chi-squared tests were used to compare the incidence rates of the outcomes of interest between groups. Logistic regression was performed as a sensitivity analysis. Statistical significance was defined at a threshold of *P* < .05. Receiver operating characteristic (ROC) curve analysis was performed, and the area under the curve (AUC) was calculated to assess the predictive ability of gHU for abnormal BMD.

**Figure 3:**
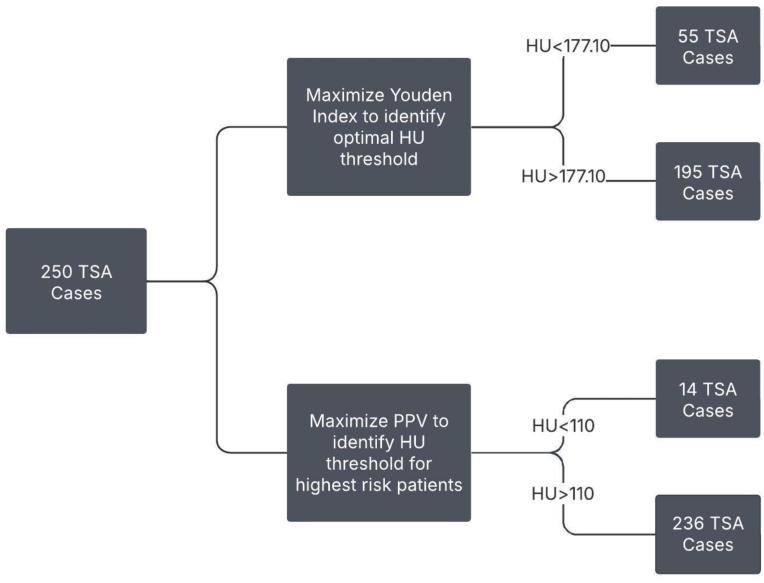
Methodology for identifying optimal gHU threshold and gHU threshold for patients at highest risk of mechanical complication after total shoulder arthroplasty. *gHU*, glenoid Hounsfield unit; *TSA*, total shoulder arthroplasty; *PPV*, positive predictive value.

Analysis was performed to determine the gHU threshold with the highest positive predictive value (PPV) for mechanical complication after TSA (Fig. 3). These patients represent the group at highest risk of mechanical complication after TSA. Since PPV is influenced by the prevalence of the condition in the population, we also maximized the Youden index value on the ROC curve to determine the optimal gHU cutoff point to separate patient risk of mechanical complications.

To determine if previously reported gHU thresholds predicted risk of complications, patients were split into 3 groups based off gHU measurement as described in Nappo et al^22^: low (gHU <197), intermediate (gHU, 197-257), and high (gHU >257). The rates of abnormal BMD within the cohort based on these same thresholds were analyzed, including all patients with CT and DEXA scans within 1 year of each other.

Inter-rater reliability was evaluated using intraclass correlation coefficients (ICCs). ICC was interpreted with 0.8-1 as excellent agreement, 0.6-0.8 as good agreement, 0.4-0.6 as moderately reliable, 0.2-0.4 as unreliable, and 0-0.2 as very unreliable.

## Results

A total of 250 TSAs in 233 total patients were reviewed. The average patient age was 67.7 years, and 64% of patients were female. Of the 250 TSAs, 180 (72%) were rTSAs. The remainder were aTSAs, except for one hemiarthroplasty. The average body mass index (BMI) of included patients was 30.87. Glenoid HU ranged from 31.7 to 795.9, with a mean of 284.4 (standard deviation = 137.1). The ICC of average gHU measurements between reviewers was calculated to be 0.896 (95% confidence interval is 0.872-0.916), suggesting excellent agreement between reviewers.

Of the 250 TSAs, there were 41 cases of mechanical complication (16.4%), eight of which resulted in revision surgery (3.2%) (Table I). There were 25 revision surgeries in total.

**Table I:** Demographic and outcome breakdown of patient cohort and groups resulting from risk stratification based on glenoid Hounsfield unit thresholds.

Group (N)	Age (SD)	Female (%)	rTSA (%)	BMI (SD)	Mechanical complication (%)	Revision (%)	Fragility fracture (%)
All patients (250)	67.70 (9.82)	160 (64)	180 (72)∗	30.87 (7.78)	41 (16.4)	25 (10)	25 (10)
Comparison: gHU <177.1 vs. >177.1							
gHU <177.1 (55)	66.56 (9.37)	40 (72.73)	49 (89.10)∗	28.97 (8.67)	16 (29.1)	6 (10.91)	7 (12.73)
gHU >177.1 (195)	68.04 (9.94)	120 (61.54)	131 (67.18)∗	31.39 (7.45)	25 (12.8)	19 (9.74)	18 (9.23)
*P* value	.31	.17	.005†	.04†	χ^2^ = 0.008; LR = 0.02†	>.99	.58
Comparison: gHU <110 vs. >110							
gHU <110 (14)	69.36 (9.15)	12 (85.71)	14 (100)∗	29.97 (9.14)	8 (57.1)	1 (7.14)	4 (28.6)
gHU >110 (236)	67.60 (9.87)	148 (62.71)	166 (70.23)∗	30.92 (7.71)	37 (14.0)	24 (10.17)	21 (8.90)
*P* value	.52	.15	.053	.66	χ^2^ < 0.001; LR = 0.002†	>.99	.06

*LR*, logistic regression; *SD*, standard deviation; *rTSA*, reverse total shoulder arthroplasty; *BMI*, body mass index; *gHU*, glenoid Hounsfield unit.

∗ Indicates the inclusion of 1 hemiarthroplasty.

† Indicates *P* < .05.

**Figure 4:**
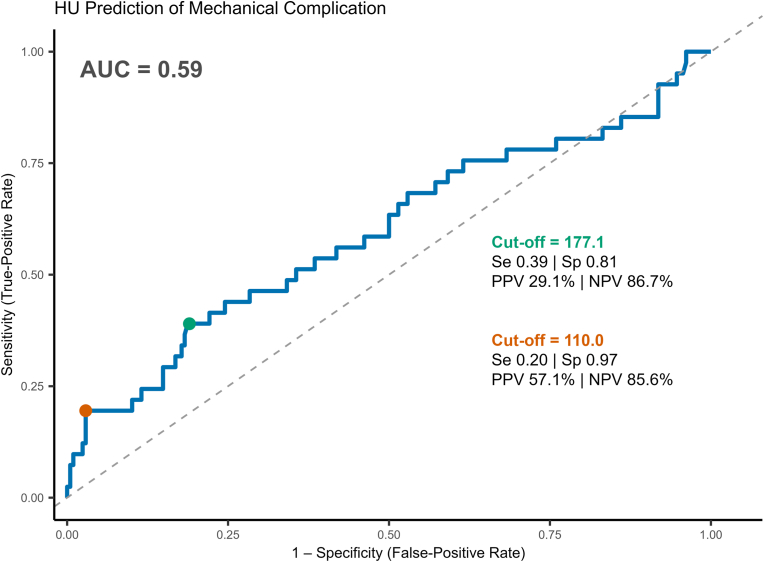
Receiver operator characteristic curve predicting mechanical complications after TSA from glenoid Hounsfield units. *AUC*, area under the curve; *HU*, Hounsfield unit; *PPV*, positive predictive value; *NPV*, negative predictive value.

The gHU threshold that stratifies patients between low and high risk for mechanical complications after TSA, the Youden threshold, was established using the ROC curve. The AUC was 0.59 and the optimal cutoff point was determined to be 177.10, with a sensitivity of 0.39, specificity of 0.81, PPV of 29.1%, and negative predictive value (NPV) of 86.7%. The threshold that maximized the PPV of gHU for complication rate, the PPV-maximizing threshold, was determined to be gHU <110 and identifies the patients at highest risk of complication after TSA (Fig. 4).

### Evaluating predictive ability of the Youden threshold glenoid Hounsfield unit <177.10 for post-operative complications

The mechanical complication rate after TSA for patients below the Youden threshold (gHU <177.1) was 29.1% (16/55 patients) and 12.8% for patients above the Youden threshold (gHU >177.1) (25/195 patients) (*P* = .008). This difference remained significant after correcting for age, gender, BMI, and type of TSA using logistic regression (*P* = .02).

Patients below the Youden threshold underwent revision surgery for any reason at a rate of 10.91% (6/55), while patients above the Youden threshold underwent revision surgery at a rate of 9.74% (19/195) (*P* > .99).

### Evaluating predictive ability of the positive predictive value–maximizing threshold glenoid Hounsfield unit <110 for post-operative complications

Rates of mechanical complication were 57.1% (8/14) for patients with gHU below the PPV-maximizing threshold and 14.0% (33/236) for patients with gHU above the threshold (*P* < .001). This relationship remained significant after logistic regression adjusting for age, gender, BMI, and shoulder arthroplasty type (*P* = .002).

The predictive ability of the PPV maximizing gHU threshold for revision surgery was evaluated. Patients with gHU below the threshold underwent revision surgery for any reason at a rate of 7.14% (1/14) compared to 10.17% (24/236) (*P* > .99) in patients above the threshold.

### Replicating the accuracy of previously reported glenoid Hounsfield unit thresholds for predicting abnormal BMD

In the low gHU group (gHU <197), the PPV for abnormal BMD was 91.67% (11/12), and in the high gHU group (gHU >257), the NPV was 60% (5/10). ROC analysis yielded an AUC of 0.71 (Table II).

**Table II:** Predictive value of glenoid Hounsfield units for abnormal bone mineral density based on previously reported thresholds.

Group	Predictive value for abnormal BMD
gHU <197 DEXA w/in 1 yr of CT	PPV = 91.67% (11/12)
gHU >257 DEXA w/in 1 yr of CT	NPV = 60% (5/10)

*gHU*, glenoid Hounsfield unit; *PPV*, positive predictive value; *NPV*, negative predictive value; *DEXA*, dual energy x-ray absorptiometry; *CT*, computed tomography.

In 25 patients with DEXA scans within 1 year of their CT scan, we found that gHU were positively correlated with lumbar spine t-scores (*P* = .04) but not proximal femur (*P* = .93) or total hip t-scores (*P* = .34).

Patients below the Youden threshold experienced fragility fractures at a rate of 12.73% (7/55), while patients above the Youden threshold experienced fragility fractures at a rate of 9.23% (18/195) (*P* = .58). Patients with gHU above the PPV threshold experienced fragility fractures at a rate of 28.57% (4/14), while 8.90% of patients below the threshold experienced fragility fractures (21/236) (*P* = .06).

## Discussion

In this study, patients with gHU <177.1 were at 227% elevated risk of mechanical complication after or during TSA compared with those above that threshold. Patients with gHU <110 were at the highest risk with our data showing a 408% increase of risk compared to patients with gHU above 110. These findings suggest that gHU from pre-operative CT can potentially be used in risk stratification of patients undergoing TSA to identify those at higher risk of mechanical complication.

Previous work has identified the association of lower HU with increased complication rates after many orthopedic procedures, including TAA, THA, spinal instrumentation, and fixation of femoral and humeral fractures.^7^^,^^11^^,^^13^^,^^15^^,^^16^^,^^18^^,^^23^^,^^24^^,^^29^ Fisher et al reported lower mean humeral HU is associated with increased risk of periprosthetic fracture after open reduction internal fixation for proximal humerus fracture. Nishi et al similarly reported increased risk of periprosthetic fracture after THA for patients with lower acetabular HU.^13^^,^^23^ Fan et al^11^ reported that lower HU of the femoral head was a risk factor for hardware failure after intramedullary nailing for intertrochanteric femur fractures. Our results are consistent with previous reports correlating lower HU with greater complication rate and suggest a cutoff of gHU <177.10 as the optimal threshold for stratifying patients into high and low risk of mechanical complication after TSA.

The cutoff of gHU <177.10 is relatively consistent with the threshold reported by Nappo et al of gHU <197 for identifying patients with abnormal BMD. Nappo et al^22^ reported two thresholds, 197 and 257. In their study 97% of patients with gHU <197 had an abnormal BMD, while 100% of patients with gHU >257 had a normal BMD. Patients with gHU between the two were of uncertain BMD status^22^ Using the same thresholds from Nappo et al, this study's population had complication rates after TSA of 25.4% (17/67) for patients with gHU <197 compared to those with gHU = 197-257 and >257 (*P* = .07). These results suggest that there may be a similar gHU risk stratification between abnormal BMD and risk of mechanical complication after TSA (Table III).

**Table III:** Complication rates in patients separated by glenoid Hounsfield Units described in Nappo et al 2018.

Group	Complication rate (*P* = .07)
gHU <197	25.4% (17/67)
gHU = 197-257	13.0% (7/54)
gHU >257	13.2% (17/129)

*gHU*, glenoid Hounsfield unit.

This study's identified threshold of gHU <177.10 is comparable to previously reported HU thresholds for predicting mechanical complications after spine and ankle procedures.^7^^,^^15^ Cody et al^7^ found that tibial HU was associated with rates of periprosthetic fracture, with HU <200 being the biggest risk factor for intraoperative periprosthetic fractures. Lee et al^15^ found that patients with L4 vertebral body HU <127.273 were at higher risk of mechanical failure after spinal instrumentation for metastatic spinal tumors, with sensitivity of 69.6%, specificity of 73.6%, PPV of 45.7%, and NPV of 88.3%.

Our analysis further supports the predictive ability of HU for post-operative outcomes in orthopedic procedures and identifies specific risk thresholds in TSA patients. Overall, this study's sensitivity (39%), specificity (81%), PPV (29.1%), and NPV (86.7%) are lower than those reported by Lee et al, suggesting that gHU may be less predictive of mechanical complication after TSA than L4 vertebral body HU are for spinal instrumentation for metastatic spinal tumors, although optimal specificity and NPV were comparable. The optimal gHU cutoff calculated (177.10) is also greater than the value Lee reported in the spine literature (127.273) but lower than that reported for TAA (200), suggesting that risk for mechanical complication after TSA may accumulate at relatively higher HU values compared to spine procedures but not TAA. Of note, the method of calculating the optimal HU threshold was identical between this study and Lee et al but differed from that of Cody et al.

Since CT scans are regularly obtained for pre-operative planning for TSA patients, the ability of gHU to predict increased risk of mechanical complications at gHU <177.10 and gHU <110 potentially allows for risk stratification at no additional cost or radiation exposure to patients. We propose that these thresholds guide pre-operative optimization, possibly including the initiation of medical treatment of osteoporosis and consideration of alternative implants or surgical techniques to mitigate risk of complications after TSA.

Beyond the primary investigation into the predictive ability of gHU for mechanical complications after TSA, the ability of gHU to predict abnormal BMD was also investigated. Previous work has reported significant predictive ability of HU from a variety of anatomical locations to predict abnormal BMD.^1^^,^^2^^,^^14^^,^^17^^,^^22^^,^^25^^,^^26^^,^^30^, ^31^, ^32^^,^^35^^,^^36^ Schreiber et al describes the association of lumbar vertebral body HU with abnormal BMD. Wagner et al reports that ulnar HU are associated with abnormal BMD. Within the shoulder literature, as detailed above, Nappo et al investigated the ability of gHU to be used for abnormal BMD screening. They suggested that patients in the “low” and “high” risk groups would not benefit from BMD screening and proposed aggressive screening for patients with gHU between 197 and 257, whose BMD status could not be predicted from gHU.

Our results are consistent with the reported ability of gHU to predict abnormal BMD but have lower accuracy metrics compared to results presented by Nappo et al. Compared to the patients included in Nappo et al, our patients were older on average (72.36 vs. 65.7) and more likely to be female (24F:1M vs. 39F:12M). A key difference between our study population and that of Nappo et al is that their cohort was drawn from a general military database, whereas our study included only patients who underwent TSA, the vast majority of whom have osteoarthritis. Our findings suggest that while gHU  <197 remains a strong predictor of abnormal BMD, these numbers are not exact and may be variable across different cohorts of patients. Regardless, the trends are similar across all studies. Our data supports abnormal BMD screening for patients with osteoarthritis and gHU >197, compared to only screening patients with gHU between 197 and 257, as Nappo proposed for the general population.

In our study, 26.8% of patients had a gHU <197. Among those patients, only 17.91% (12 out of 67) underwent a DEXA scan within 1 year, and 25.27% (17 out of 67) had a scan within two years. Given the high PPV of gHU  <197 for abnormal BMD, there is a significant opportunity to use gHU as an opportunistic screening tool to identify patients at risk of abnormal BMD. This finding is consistent with previously reported conclusions that TSA patients are under-screened for abnormal BMD.^19^

Our study has several limitations. The retrospective nature of this analysis prevents any patient randomization and causal inference. Of the 250 cases included in the study, 55 involved patients with gHU <177.10 and 14 involved patients with gHU <110. Alhough we detected a statistically significant increase in risk of mechanical complication for patients with gHU less than both thresholds, larger sample sizes would strengthen the predictive ability of gHU for complication and modify the specific threshold to optimize clinical application. Due to low sample size and low incidence rates, our study was underpowered to detect the relationship between gHU and other adverse mechanical outcomes, including t-scores (power = 27%), revision rate (power = 6%), and fragility fracture (power = 37%). Lastly, our definition of mechanical complication includes aseptic loosening, but periprosthetic joint infections of the shoulder can cause loosening of implants and often follow an indolent course. These more subtle cases of septic loosening may not have been identified and excluded from our definition of mechanical complication, especially for loosening events that did not ultimately result in revision. Future work should aim for prospective multicenter trials or large databases that include CT scans to strengthen the understanding of HU and complications in TSA and to working to develop the most effective way to mitigate risk of complication after patients are identified as high risk.

## Conclusion

Our data suggests that pre-operative glenoid neck HU measurements can be used to predict the risk of mechanical complications after TSA. Measurement of gHU exhibits high inter-rater reliability. Patients with gHU <177.10 have elevated risk, while patients with gHU <110 are at the highest risk of mechanical complications after TSA. This suggests that gHU can potentially be used for risk stratification of patients for mechanical complications after TSA using pre-operative CT. Validation of our reported gHU thresholds in larger, prospective cohorts is needed. The effectiveness of different medical, surgical, or implant-level strategies to improve outcomes for high-risk patients is an important direction for future research.

## Disclaimers

Funding: No outside funding or grants were received for any stage of research described in the present manuscript, including data collection, data analysis, and preparation of the manuscript.

Conflicts of interest: The authors, their immediate families, and any research foundation to which they are affiliated have not received any financial payments or other benefits from any commercial entity related to the subject of this article.
